# Early detection of pulmonary vasculopathy in children with sickle cell disease by new echocardiography-based blood speckle technology

**DOI:** 10.1186/s12887-025-05689-3

**Published:** 2025-05-14

**Authors:** Antoine Fakhry AbdelMassih, Fatima Nabil, Niveen Salama, Ilham Youssry

**Affiliations:** 1 Pediatric Cardiology Unit, Pediatrics Department, Faculty of Medicine, Cairo University, Cairo, Egypt; 2 Pediatric Residency Program, Faculty of Medicine, Cairo University, Cairo, Egypt; 3 Pediatric Hematology Unit, Pediatrics Department, Faculty of Medicine, Cairo University, Cairo, Egypt; 4 Pediatric Department, Pediatric Cardiology Division, Specialized Pediatric Hospital, Faculty of Medicine, Cairo University, Kasr Al Ainy Street, Cairo, 12411 Egypt

**Keywords:** Sickle cell disease, Blood speckle tracking, Vortex, Pulmonary vasculopathy

## Abstract

**Background:**

Right ventricle (RV) dysfunction because of pulmonary vasculopathy is the most common cause of death in sickle cell disease (SCD). This study aimed to explore the diagnostic accuracy of blood-speckle tracking and vortex detection in the early detection of pulmonary vasculopathy in patients with SCD.

**Methods:**

This study was conducted as a cross-sectional study including thirty patients and thirty controls. Patients with SCD were examined using 3D echocardiography to determine the presence of RV dysfunction, as a surrogate of pulmonary vasculopathy: in addition to blood speckle tracking echocardiography to determine the vortex timing in the RV and its presence or absence in the pulmonary artery. Patients’ demographic and hematologic data were also retrieved from patients’ files.

**Results:**

Pulmonary vortex formation was 100% sensitive in the detection of RV dysfunction. LDH (Lactate Dehydrogenase) was the only variable significantly different between cases with pulmonary vortex formation and those without (520 vs. 257, *P* < 0.001), LDH > 400 was 72% sensitive and 100% specific in the detection of pulmonary vortex formation.

**Conclusions:**

Pulmonary vortex formation was a sensitive indicator of RV dysfunction, thus suggesting its accuracy in the early detection of early pulmonary vascular changes in SCD. LDH as a marker of intravascular hemolysis, is a sensitive marker that can be used for risk stratification of SCD patients.

## Impact statement

This article explores the use of a novel echocardiographic technique, blood speckle tracking, for the early detection of pulmonary vasculopathy in patients with sickle cell disease (SCD).

Blood speckle tracking is a recently introduced echocardiographic technique; however, studies evaluating its role in systemic disorders remain scarce or entirely absent.

Current methods for detecting pulmonary vasculopathy are limited—cardiac catheterization is invasive, cardiac MRI is costly, and tricuspid regurgitant velocity assessment often leads to delayed diagnosis. Validating this technique could enhance the care and management of SCD patients.

## Background

Sickle cell disease (SCD) is the most common inherited hemoglobinopathy worldwide. Improvements in health infrastructure, preventive care, and clinical treatments have reduced the morbidity and mortality of sickle-cell diseases in developed countries. However, as these patients live longer, the chronic effects of sustained hemolytic anemia and episodic vaso-occlusive events drive the development of end-organ complications especially pulmonary vasculopathy and myocardial involvement because of the microvascular dysfunction accompanying SCD [1].

A single institution assessment of patients with SCD revealed that nearly 40% of premature deaths in patients were related to cardiopulmonary complications, which included myocardial infarction and congestive heart failure [2].

The five main causes of death according to death certificates were cardiovascular in 32% of patients and respiratory in 28%. Pulmonary Hypertension (PHT) in SCD ranges from 10 to 33% when measured by Right heart catheterization (RHC) or echocardiography, respectively [3].

The pathology of PHT varies and can involve pulmonary vascular endothelial dysfunction, smooth muscle cell (SMC) proliferation, adventitial fibroblast accumulation, and immune system activation. Overall, PHT generates right ventricular (RV) pressure overload, causing molecular adaptations predisposing to RV failure [4].

On the basis of the increased risks of pulmonary hypertension and death related to high tricuspid regurgitant (TR) velocity and plasma Brain Natriuretic Peptides (NT-proBNP) concentrations, the American Thoracic Society recommend annual screening of these values for SCD patients [5].

This approach relies on detecting pulmonary hypertension via echocardiographic assessment of tricuspid regurgitant velocity. However, a study by Parent et al. found that echocardiographic evaluation alone has a low positive predictive value for pulmonary hypertension.6 This limitation highlights the need for more advanced echocardiographic techniques to assess pulmonary hypertension and right ventricular dysfunction accurately. Consequently, it calls into question the reliability of this approach and underscores the need for alternative screening methods for pulmonary vasculopathy [6].

Cardiac MRI (CMR) offers itself as the gold standard in detection of myocardial affection and intracardiac blood flow abnormalities in these patients, however the cost and lengthy examination can constitute a barrier against its use [7].

Several echocardiographic techniques have been made available that can slowly replace the need for the lengthy and costly CMR and can detect the disordered myocardial deformation and the pulmonary vasculopathy in SCD patients with a promising diagnostic accuracy. Two techniques can notably achieve this diagnostic accuracy, myocardial tissue speckle tracking (3D STE) and the very newly introduced “blood speckle tracking technique” [8].

The aim of this study was early detection of pulmonary vasculopathy in patients with SCD using blood speckle tracking, also to test the effect of different independent variables such as the extent of hemolysis, and frequency of vaso-occlusive crises in exacerbation of pulmonary vasculopathy and myocardial dysfunction in those patients.

## Methods

### Study subjects

This case-control study was conducted at the Hematology Outpatient Clinic of Cairo University Children’s Hospital (CUCH) between January and June 2022. It included two groups:

#### Group 1

This group comprised 30 patients with a confirmed diagnosis of sickle cell disease (SCD), verified by the presence of HbS as measured by high-performance liquid chromatography [9]. These patients were regularly monitored at the Hematology Clinic of Cairo University Children’s Hospital. Exclusion criteria included any known structural heart disease, except for patent foramen ovale (PFO) or atrial septal defect (ASD) that did not lead to right ventricular (RV) dilation.

#### Group 2

This group included 30 healthy controls matched for age, sex, and body surface area, ***who served solely as a benchmark for echocardiographic data***. These individuals had no history of chronic illnesses and were recruited from the same outpatient clinic after thorough assessments to eliminate any significant acute or chronic medical conditions. Informed consent was obtained from the guardians of all participants before the collection of data and samples. The study protocol received approval from the Pediatrics Department at the Faculty of Medicine, Cairo University.

### Study methods

#### Revision of the patient files for the following data

*Demographic characteristics such as age, sex, weight, and surface area.

*Clinical characteristics: age at first blood transfusion, volume of annual blood transfusion, frequency of vasocclusive crises.

*Laboratory characteristics: last hemoglobin level, reticulocytic count, serum Lactate dehydrogenase (LDH) concentration and serum ferritin, total and direct bilirubin before echocardiographic examination.

#### Advanced echocardiographic examination

*Conventional and Tissue Doppler:

Peak early diastolic tissue velocity (E′) was measured at the septal and lateral mitral annulus. Mitral inflow velocity was assessed using pulsed-wave Doppler from the apical four-chamber view, with the sample volume positioned at the mitral leaflet tips. The E/E′ ratio was derived by dividing the E wave velocity by the corresponding e′ velocities.”

*3D Echocardiography:

Echocardiographic recordings were obtained using General Electric (GE) E95 ultrasound machine (GE Vingmed Ultrasound, Horten, Norway-2015).

- 3D Echocardiography: full-volume acquisition of the Left Ventricle (LV) was obtained by harmonic imaging from the apical approach. Three ECG-gated consecutive beats were acquired during end-expiratory breath-hold to LV full volume. The depth and volume size were adjusted to obtain a temporal resolution higher than 30 volumes/s. All data sets were analyzed off-line using the GE built-in software. The software automatically identifies the LV cavity endocardial border in 3D. The operator were perform all the necessary adjustments manually in order to correctly place the endocardial border. Papillary muscles were included within the LV Chamber. The software then automatically calculates the left ventricular end-diastolic volume (EDV), ejection fraction (EF), left ventricular global longitudinal, area and circumferential strains (GLS, GAS, GCS respectively).
- 3DE RV datasets were digitally analyzed offline using the GE built-in software to generate RV functional indices automatically: 3D end-diastolic volume (EDV), 3D end-systolic volume (ESV), 3D stroke volume (SV), 3D EF, fractional area change and tricuspid annular plane systolic excursion. (TAPSE)

>> Blood speckle tracking (BST): All the studied patients underwent a complete 2D and color doppler echocardiography and a further Blood Speckle Imaging evaluation. For BST analysis, we used 6 S probe and a frame per second (FPS) ranging between 400 and 500 [10]. Data were acquired while in conventional color Doppler mode at a depth < 10 cm and using a 60 cm/s color scale velocity. The BST loops were acquired at very high frame rate and then recalled showing the blood flow trajectories. The images were analyzed by two blinded physicians to determine the timing of vortex formation in the right ventricle and the absence or presence of vortex formation in the pulmonary artery. The occurrence of vortex in the pulmonary artery as well as a late diastolic vortex in the right ventricle were considered pathologic [11].

### Statistical analysis

As considered the primary outcome, sample size calculation was done using the comparison of the occurrence of abnormal pulmonary artery vortex, by CMR between children with pulmonary hypertension and matched healthy children. Calculation was done based on comparing two proportions from independent samples in a prospective study using Fischer exact test. The α-error level was fixed at 0.05, The power was set at 80%, and the case-control ratio was set. According to Mawad et al. findings [12], 80% of pulmonary hypertension patients have a pulmonary artery vortex, while all normal children have no pulmonary vortex. Accordingly, the minimum optimum sample size should be 23 children in each group to detect a real difference of 30% in pulmonary artery vortex. Sample size calculation was done using PS Power and Sample Size Calculation software, version 3.0.11 for MS Windows (William D. Dupont, and Walton D, Vanderbilt University, Nashville, Tennessee, USA) [13].

Accordingly, our sample will include 30 sickle cell anemia patients and 30 controls.

Data were analyzed using Medcalc statistical software, numerical data were tested for normality of distribution using Shapiro-Wilk test, normally distributed data were represented using mean and standard deviation (SD), while skewed data were expressed as median and interquartile range (IQR), and compared using independent samples T-Test. Categorical variables were expressed as number and percentages to the total and compared using either Chi square or Fisher testing when applicable. A receiver operating characteristic (ROC) analysis was performed to test the diagnostic accuracy of abnormal RV vortex and pulmonary vortex formation in predicting RV systolic dysfunction (as defined by RV EF < 40%) and to measure the cut-off LDH level in predicting pulmonary vortex formation.

## Results

Table 1 presents demographic and hematologic data for studying subjects. The age range of the cases was between 5 and 18 years, which was comparable to the control group, whose age ranged from 5 to 17 years. Statistical analysis revealed no significant differences between the cases and controls in terms of age and sex distribution. The median age of start of blood transfusion was 1.5 years, and the median Hb was 8.6. The median serum ferritin was 370, while the median LDH was 400.

**Table 1:** Demographic data of cases vs. controls

		Cases (*n* = 30)	Controls (*n* 30)	*P* value
Age	(years) mean ± SD	9.87 ± 3.40	9.16 ± 2.6	0.273
	Median (min-max)	10 (5–18)	9 (5–17)	
Weight (kg) mean ± SD		26.77 ± 8.87	26.77 ± 6.28	1.000
Surface area (m^2^) mean ± SD		0.90 ± 0.21	0.91 ± 0.17	0.893
Serum ferritin (ng/dL) median (IQR)		370 (135–900)		
LDH (units/l) median (IQR)		400 (251–583)		
Reticulocytes (%) median (IQR)		5.9 (2-8.3)		
Total bilirubin (mg/dl) median (IQR)		1.8 (1.2–2.3)		
Direct bilirubin (mg/dl) median (IQR)		0.4 (0.3–0.5)		
Hemoglobin (gm/dl) median (IQR)		8.65 (7-10.4)		
Number of VOC/year median (IQR)		3 (1–5)		
Volume of blood transfusion /year (ml/kg) median (IQR)		30(15–60)		
Age of start of blood transfusion (years) median (IQR)		1.5 (1–2)		

Abbreviations: dl: deciliter, Kg: Kilogram, IQR: interquartile range, LDH: Lactate dehydrogenase, mg: milligram, ml: milliliter, n: number, SD: standard deviation, VOC: vasocclusive crises

Echocardiographic data between cases and controls, listed in Table 2, showed evidence of significant RV and LV dysfunction in cases compared to controls. LV GLS (%) was markedly reduced in cases 21 ± 4 vs. 24 ± 2 in controls. RV indices derived from 3D echocardiography showed that RV EF (%) is significantly lower in cases 38 ± 3 vs. 66 ± 1.9 in controls.

**Table 2:** Echocardiographic data of cases vs. controls

		Cases (*n* = 30)	Controls (*n* = 30)	P value
		Mean ± SD	Mean ± SD	
Left ventricular data	GLS (%) mean ± SD	21.83 ± 4.84	24.43 ± 2.36	0.011*
	GCS (%) mean ± SD	14.10 ± 3.18	24.63 ± 2.40	< 0.001*
	GAS (%) mean ± SD	30.07 ± 5.32	38.47 ± 3.00	< 0.001*
	Twist (°) mean ± SD	4.23 ± 4.37	6.67 ± 1.47	0.006*
	Fractional area change (%) mean ± SD	32.03 ± 9.72	37.67 ± 5.03	0.007*
	LV EDV (ml) mean ± SD	64.77 ± 9.48	70.00 ± 4.42	0.009*
	LV EF (%) mean ± SD	57.63 ± 10.25	71.83 ± 3.89	< 0.001*
	LV sphericity index mean ± SD	0.47 ± 0.08	0.36 ± 0.02	< 0.001*
	LV E/E’ mean ± SD	11.90 ± 2.82	4.97 ± 1.56	< 0.001*
Right ventricular data	RV EDV (ml) mean ± SD	50.47 ± 14.78	49.17 ± 3.77	0.643
	RV EF (%) mean ± SD	38.43 ± 9.11	66.10 ± 1.95	< 0.001*
	RV TAPSE (mm) mean ± SD	16.37 ± 6.05	19.23 ± 3.86	0.033*
	No TR	3 (10%)	4 (13%)	0.17
	Incomplete Doppler Window	12 (40%)	18 (60%)	
	Well visualized TR doppler window	15 (50%)	8 (27%)	
	RVSP from TR (mmHg) mean ± SD	22 ± 3	22 ± 3.65	0.624
	Pathological RV (systolic) vortex n (%)	15 (50.0%)	3 (10.0%)	0.001*
	Physiological RV (diastolic) vortex n(%)	15 (50.0%)	27 (90.0%)	
	Present pulmonary artery vortex n (%)	18 (60.0%)	1 (3.3%)	< 0.001*
	Absent pulmonary artery vortex n (%)	12 (40.0%)	29 (96.7%)	

Abbreviations EDV: End-diastolic volume, EF: ejection fraction, GAS: Global area strain, GCS: Global circumferential strain, GLS: global longitudinal strain, LV: left ventricle, RV: right ventricle, RVSP: Right ventricular systolic pressure, TAPSE: tricuspid annular plane systolic excursion

**Fig. 1:**
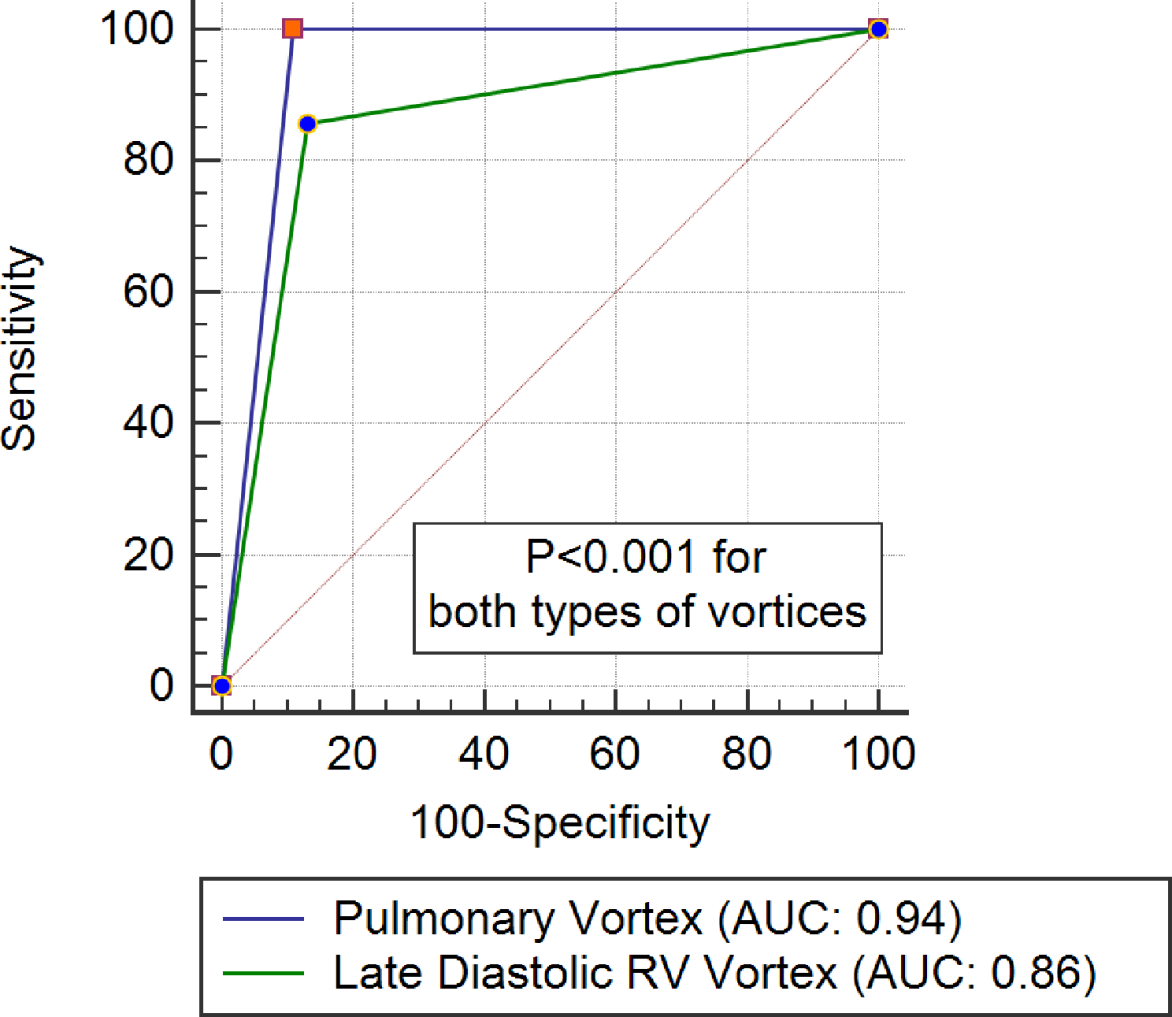
Receiver operating characteristic curve to show the diagnostic accuracy of RV systolic vortex and pulmonary vortex formation in detection of RV ventricular dysfunction (RV EF < 40%)

We investigated in Fig. 1 the diagnostic accuracy of pulmonary and systolic RV vortex formation as well as conventional RV systolic pressure derived from TR, in predicting RV dysfunction in pulmonary vasculopathy, and a cut-off of 40% was suggested for normal RV function. The ROC analysis showed that RV systolic vortex and pulmonary vortex formation were highly sensitive markers of RV dysfunction (Sensitivity 100% and 85% /AUC 0.94 and 0.86 respectively, P<0.001)

Pulmonary vortex formation was seen in only one control subject, while 60% of cases showed pulmonary vortex formation. RV systolic vortex formation was seen in 10% of controls vs. 50% of cases. (Table 2). Patients were then divided according to the presence or absence of a pulmonary vortex into two groups, laboratory, and hematologic data (Table 3) were compared between the subgroups of cases. Serum LDH was significantly higher in patients with pulmonary vortex formation.

**Table 3:** Hematologic data in subgroups of cases based on pulmonary vortex formation

	Pulmonary artery vortex		*P* value
	Present (*n* = 18)	Absent (*n* = 12)	
Age (years) mean ± SD	9.44 ± 3.45	10.50 ± 3.37	0.415
Sex (%)	26.39 ± 10.19	27.33 ± 6.80	0.781
Body Surface area (m^2^)	0.89 ± 0.24	0.92 ± 0.18	0.670
Serum ferritin (ug/l) mean ± SD	370.00 (117–648)	523.00 (172–1345)	0.346
LDH (units/l) mean ± SD	520.50 (400–653)	257.00 (225–372)	< 0.001*
Reticulocytes (%) Median (IQR)	7.25 (2–9)	2.70 (2.15–6.9)	0.415
Total bilirubin (mg/dl) Median (IQR)	1.75 (1.2–2.2)	1.90 (1.3–2.35)	0.491
Direct bilirubin (mg/dl) Median (IQR)	0.30 (0.29–0.4)	0.40 (0.35–0.6)	0.134
Hemoglobin (gm/dl) Mean ± SD	8.73 ± 0.61	8.71 ± 1.09	0.950

Abbreviations: IQR: interquartile range, LDH: Lactate dehydrogenase, n: number

**Fig. 2:**
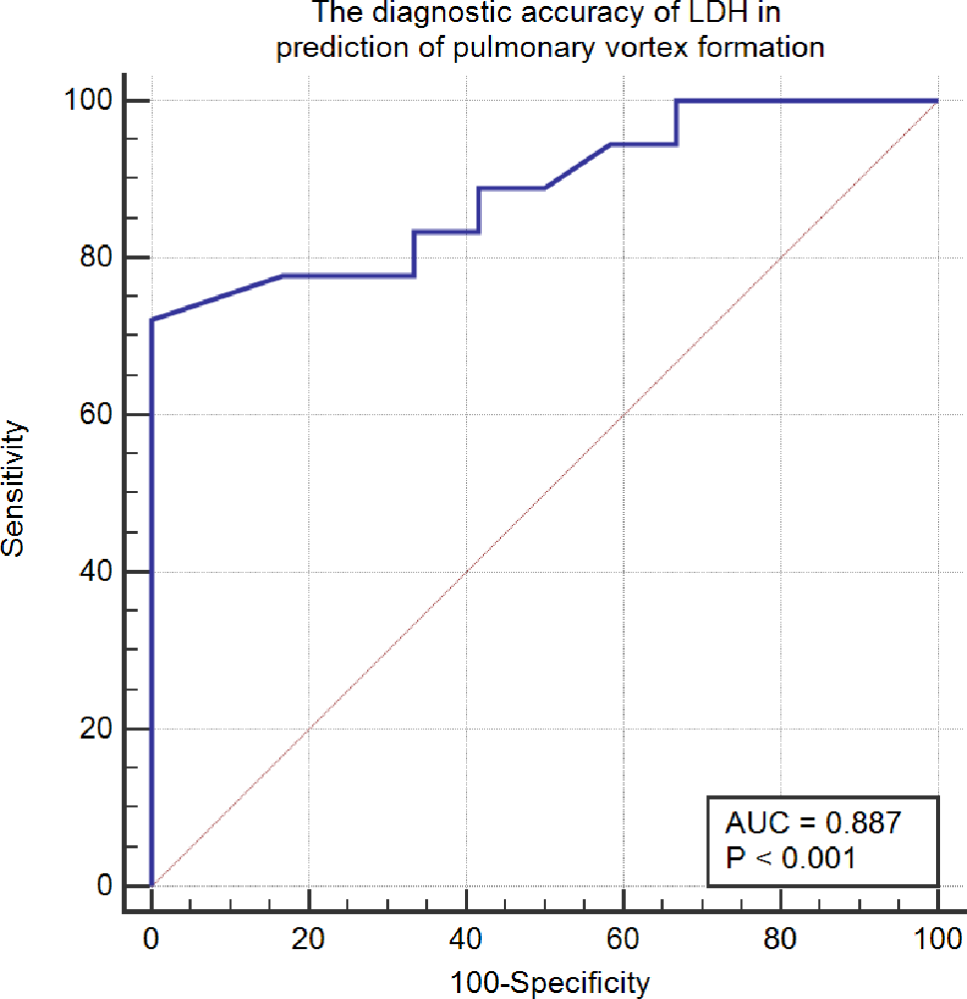
Receiver operating characteristic curve to reflect the diagnostic accuracy of LDH in detection of pulmonary vortex formation

LDH > 400 was 72% sensitive and 100% specific of pulmonary vortex formation (AUC: 0.887, *P* < 0.001) (Fig. 2).

**Fig. 3:**
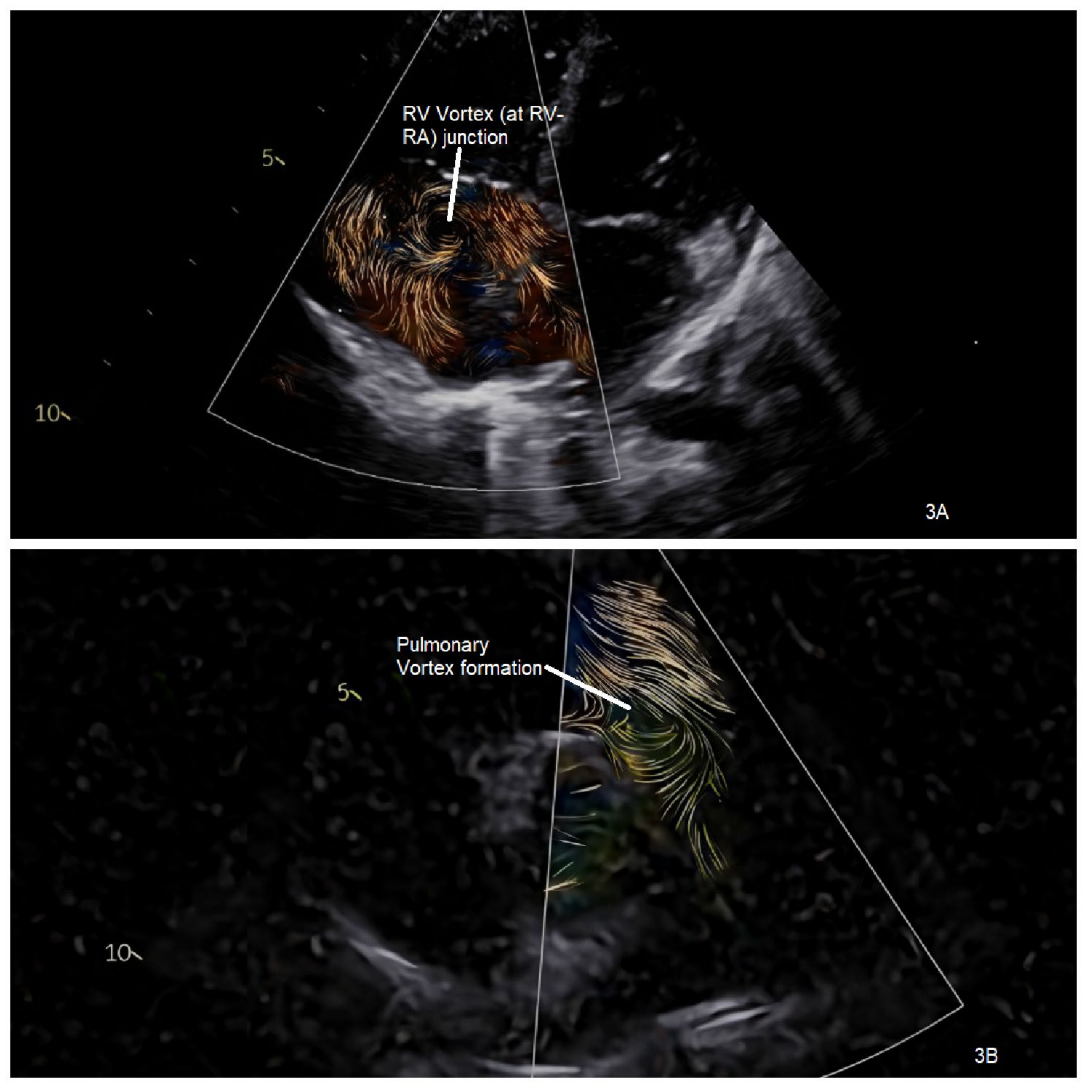
**A**/**B**: RV vortex formation/ Main Pulmonary artery vortex formation

Figure 3A and B are two samples of RV and Pulmonary vortex formations from our study subjects.

## Discussion

This study explored the ability of echocardiography-mediated blood vortex assessment and timing to detect possible pulmonary vasculopathy. Pulmonary vortex formation was seen in 60% of cases also achieving high sensitivity (AUC: 0.9) in predicting RV dysfunction. RVSP deducted from TR jet was not significantly different between cases and controls and was less sensitive in detecting RV systolic dysfunction (AUC: 0.5, *P* = 0.3).

Intracardiac and intravascular blood flow abnormalities, could be an early sign of myocardial involvement and of vasculopathy, and implementing the new technique of blood speckle tracking can be a game-changer in early detection of pulmonary vasculopathy in SCD patients [14, 15].

The use of vortex detection in diagnosing pulmonary hypertension (PHT) represents a promising frontier in cardiopulmonary diagnostics. An elevated mean pulmonary arterial pressure (mPAP) often leads to vortical blood flow patterns in the main pulmonary artery. This phenomenon is not just a curious hemodynamic alteration; it suggests underlying pathological changes that could assist in the early detection and monitoring of PHT. In a study by Mawad and colleagues, 80% of patients showed abnormal pulmonary artery vortex compared to 0% of controls [12, 16].

Also, filling with maximal velocity and subsequent vortex formation in the ventricle are seen usually in early (E) phase of diastole. Late diastolic (A) extending to systolic vortices have been linked to diastolic dysfunction of RV that is commonly encountered in pulmonary vasculopathy. In this study we were able to reproduce a similar finding by demonstrating a predominance of RV late diastolic-systolic vortices compared to an early diastolic vortex in controls. These findings signify that maximal filling of the RV is shifted in SCD patients from early diastole to late diastole and systole (A and S phases), which can suggest the occurrence of ventricular dyssynchrony due to pressure load from pulmonary vasculopathy [11, 17, 18].

Previous studies in the same context showed Late diastolic (A) extending to systolic vortices linked to diastolic dysfunction of RV that is commonly encountered in pulmonary vasculopathy. In another report, using contrast magnetic resonance imaging, fifteen subjects, nine with PAH and six healthy volunteers, underwent 4D-flow. The study confirmed that abnormal blood flow dynamics, including the vortex formation in the PA, were characteristics of vaso-occlusive pulmonary hypertension. The latter mechanism is the main pathogenesis involved in induction of pulmonary hypertension in sickle cell disease [11, 14].

These findings were associated with RV dysfunction, which might be suggestive of early pulmonary vasculopathy. The term vasculopathy is a broader term, which encompasses different degrees of alterations in the pulmonary arterioles, up to the development of pulmonary hypertension. The reason behind using this terminology in our study instead of pulmonary hypertension, is that we were not able to measure accurately pulmonary artery pressure and pulmonary vascular resistance invasively by right heart catheterization [19].

The presence of vortical flow patterns can serve as an indirect marker for increased mPAP. As blood encounters resistance due to heightened vascular tone or structural alterations in the pulmonary vasculature, the resulting flow dynamics may change, allowing clinicians to identify vasculopathy even before significant symptoms emerge and prior to the development of significant changes in RVSP as measured by TR. To our knowledge this is he first study on intracardiac blood flow abnormalities on SCD patients by the echo-based technique.

Moreover, the ability to detect and analyze vortices could potentially enhance risk stratification and guide more personalized treatment approaches. There is still conflicting evidence regarding the role and significance of pulmonary vortices in pulmonary hypertension. While some studies suggest their importance, as mentioned earlier, others downplay their role. The lack of a universally accepted definition and difficulties in accurately visualizing and quantifying vortices in vivo contribute to this conflict [11, 20, 21].

The development of early pulmonary vasculopathy in SCD, is mediated via an interplay between oxidative stress, endothelial dysfunction coagulopathy, sickling creating a vicious circle that promote end-organ damage [22–25].

In this study, the best predictor of RV dysfunction and pulmonary vortex formation was serum LDH, while the hemoglobin concentration, reticulocytic count and frequency of vasocclusive crises were not correlated with echocardiographic evidence of pulmonary vasculopathy. LDH presents itself as a marker of arterial stiffness and microvascular dysfunction; a relatively recent study by Zhu et al. showed that LDH level > 172 U/L is associated with increased cardiovascular risk. Moreover, LDH is regarded as a marker of hemolysis-associated NO resistance phenotype of sickle cell disease, endothelial dysfunction, and end-organ vasculopathy.

## Study limitations

The primary limitations of this study arise from the qualitative nature of the currently available software for vortex detection via echocardiography. While this software facilitates the visualization of vortices, it falls short in accurately quantifying blood velocity and the diameters of these vortices. Given the extensive range of measurements that cardiac MRI can provide in similar contexts, there is still significant progress to be made in enhancing the capabilities of echocardiographic vortex analysis.

Another limiting factor is the absence of invasive hemodynamics, to benchmark BST results to it.

## Conclusions

Blood speckle tracking is a new technique that has proven sensitivity in detection of early pulmonary vascular changes. It has been made available using CMR and 4D flow analysis and achieved in previous recent works good sensitivity in diagnosis of pulmonary hypertension compared to invasive methods. This is to our knowledge the first study to employ an echocardiography-based software to estimate its accuracy by diagnosing alterations in pulmonary arteries in the context of SCD. Despite being exclusively qualitative, the new modality, showed significant difference between cases and controls regarding pulmonary and right ventricular vortex formation. Larger studies might be needed to generalize the findings of the study, also improving the modality by adding some functional parameters (vortex maximal diameter and accurate timing in relation to aortic valve closure) will render it truly applicable in clinical day to day setting.

## Data Availability

Data underlying this manuscript will be provided upon reasonable request.

## Abbreviations

ASD: Atrial septal defect
AUC: Area under the curve
BNP: Brain Natriuretic Peptide
BST: Blood speckle Tracking
CMR: Cardiac Magnetic resonance
ECG: Electrocardiogram
EDV: End-diastolic volume
EF: Ejection Fraction
FPS: Frame per second
GAS: Global Area strain
GCS: Global Circumferential Strain
GE: General Electric
GLS: Global longitudinal strain
LDH: Lactate dehydrogenase
LV: Left Ventricle
LV E/E': Ratio of early mitral inflow velocity to average early diastolic velocities of the mitral annulus and basal septum
mPAP: mean pulmonary artery pressure
MS: Microsoft
PAH: Pulmonary artery pressure
PHT: Pulmonary Hypertension
ROC: Receiver Operating characteristic
RV: Right ventricle
SCD: Sickle cell disease
STE: Speckle Tracking echocardiography
SV: Stroke volume
TAPSE: Tricuspid annular plane systolic excursion
TR: Tricuspid regurge
